# Keeping NO in No-Touch Saphenous Vein Bypass Grafts

**DOI:** 10.21470/1678-9741-2025-0040

**Published:** 2025-11-17

**Authors:** Michael R. Dashwood

**Affiliations:** 1 Surgical and Interventional Sciences, Royal Free Hospital Campus, University College Medical School, London, United Kingdom

**Keywords:** Saphenous Vein, Coronary Artery Bypass, Nitric Oxide, Patency.

## Abstract

The saphenous vein is the most commonly used conduit in patients undergoing coronary artery bypass surgery. Graft patency is improved using the no-touch technique where the vein is harvested with minimal trauma, avoiding high pressure distension and maintaining normal vessel architecture. Various cells that are damaged when using conventional harvesting are preserved using the no-touch technique and are a source of nitric oxide. The no-touch technique is becoming accepted more widely as is the role of nitric oxide in improved saphenous vein graft patency. However, there are conflicting views regarding the tissue sources of nitric oxide.

## INTRODUCTION

**Table:** 

Abbreviations, Acronyms & Symbols	
A	= Adventitia
CT	= Conventional
eNOS	= Endothelial nitric oxide synthase
L	= Lumen
M	= Media
NO	= Nitric oxide
NOS	= Nitric oxide synthase
NT	= No-touch
PVAT	= Perivascular adipose tissue
PVF	= Perivascular fat
SV	= Saphenous vein

**Fig. 1:**
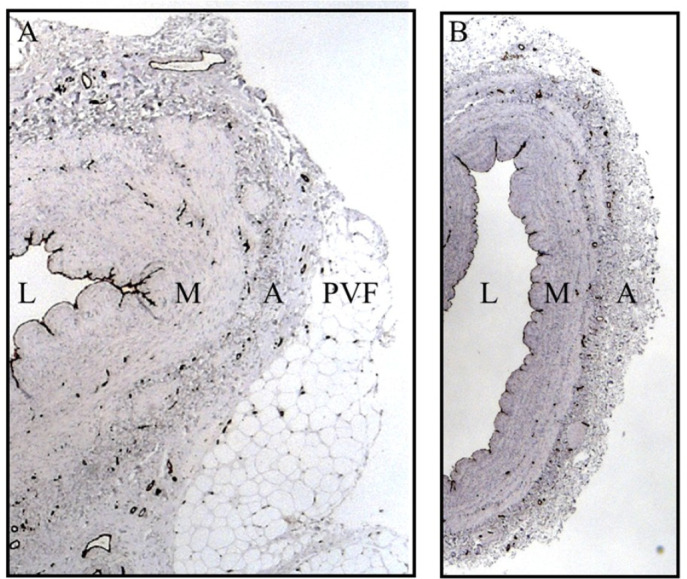
Endothelial cells of no-touch and conventional saphenous vein grafts. A) Part of transverse section of a no-touch saphenous vein showing endothelial cells identified using CD31 (dark immunostaining). The lumen (L) is folded as no distension is used and shows a continuous/undamaged endothelial lining. Endothelial cell staining of vasa vasorum is located in the media (M) and adventitia (A), extending to capillaries within the perivascular fat (PVF). All endothelial cells express positive endothelial nitric oxide staining. B) Part of transverse section of a conventional saphenous vein. L is dilated due to high pressure distension and regions of the endothelium are damaged. M is thin, the outer part of A is damaged and the density of vasa vasorum is reduced when the PVF is removed at harvesting. Endothelial cells express positive endothelial nitric oxide staining.

The saphenous vein (SV) is the most commonly used bypass graft for myocardial revascularization in patients with coronary artery disease, and the role of perivascular fat in improving graft performance has been described in a previous issue of the Brazilian Journal of Cardiovascular Surgery^[1]^. The recognition that nitric oxide (NO) plays a pivotal role in improved SV graft patency has been reported in two recent publications^[2,3]^. In the most recent article, *Can a Frog Become a Princess?*, the authors conclude “…in our story, the recently discovered NO seems to play a key role, not only in increasing the rank of SV graft [patency]….”^[3]^. This is hardly a new finding given the established protective role of endothelium-derived NO on the vasculature. In the Frog to Princess Hypothesis, much discussion is based on the identification of nitric oxide synthase (NOS) in SV harvested with minimal trauma using the no-touch (NT) technique where vascular and endothelial damage is reduced compared with conventionally harvested SV^[4,5]^. This observation was first reported in an early scanning electron microscope study that revealed a reduction in the area of the luminal endothelium of conventional (CT) *vs.* NT SV grafts^[4]^. Endothelium-dependent NOS was identified on sections of NT and CT SV, and the reduction of endothelial cells (73% NT *vs.* 52% CT; *P* = 0.04) was associated with a concomitant reduction of endothelium-dependent NOS staining in both the lumen and vasa vasorum of CT *vs.* NT SV^[5]^. Interestingly, although endothelial localization of NOS was anticipated, there was also dense staining of other cells in SV sections, including vascular smooth muscle cells in the tunica media and endothelial cells of the vasa vasorum. However, as the methods used in this study were not isoform-specific it is possible that multiple NOS isoforms (endothelial NOS [eNOS], inducible NOS, and neuronal NOS) may exist throughout the SV wall. Therefore, a subsequent study specifically examined the distribution of eNOS in histological sections of NT and CT SV grafts using immunohistochemistry with eNOS protein expression assessed in tissue extracts by western blot analysis. NOS activity, as an indicator of NO production, was also measured using the citrulline assay^[6]^. On microscopic examination, considerable vascular injury to CT *vs.* NT SV sections was observed. There was also damage to the outer adventitia caused at harvesting due to the removal of perivascular adipose tissue (PVAT) of CT SV used as bypass grafts (Figure 1). eNOS immunostaining was absent in regions of endothelial denudation and also reduced due to damage of the adventitia and vasa vasorum of CT *vs.* NT SV sections^[7,8]^. This reduction of eNOS in histological sections was supported by a highly significant reduction in both eNOS protein expression (*P* < 0.0001) and NO production (*P* < 0.0001) in tissue extracts of CT *vs.* NT SVs^[6]^. These results imply that the reduced eNOS/NO release in SVs harvested by CT surgery compared with those prepared by the NT technique influences graft performance^[6,8]^.

## COMMENTS

Problems associated with endothelial damage of SV grafts are well documented^[6]^. A variety of solutions used for storing SV explants in the operating room after harvesting have been described possessing anti-contractile effects that have both earlyand long-term beneficial effects on SV graft patency. Such solutions range from autologous heparinised blood and heparinised normal saline to University of Wisconsin preservation solution and glyceryl trinitrate-verapamil solution^[9]^. The main role of these solutions is to provide early protection of the SV endothelium. A recent study comparing the effect of two preservation solutions on isolated SV endothelial cells and on histological sections of patients’ SV grafts has shown that the endothelial damage inhibitor, DuraGraft®, is more effective than commonly used full electrolyte solution^[10]^. Here, measuring percent luminal endothelium on sections of SV, DuraGraft® solution provided superior protection compared with full electrolyte solution (74 ± 8% *vs.* 56 ± 8%, *P* < 0.001). These results support the suggestion that short-term storage of SV grafts in endothelial damage inhibitors at harvesting preserves endothelium/endothelium-dependent NO release and improves SV graft patency.

Apart from the endothelium, other potential sources of NO include the vasa vasorum, PVAT, and its capillary network, all structures that remain intact in NT SV but are removed or damaged in CT SV grafts (Figure 1)^[5,6]^. While Calafiore et al. concur regarding the role of the luminal endothelium as a source of NO, they reject the role of perivascular fat, suggesting that “…with the no-touch technique, this aspect is not obvious, as it is not certain that the NO produced by the perivascular adipose tissue can reach the lumen of the SVG”^[3]^. This is surprising since, in their *Increased Nitric Oxide Availability* article, the same authors state that “Perivascular adipose tissue produces nitric oxide via endothelial nitric oxide synthase and exerts a paracrine effect on the adjacent vasculature”. Also, apart from the role of endothelium and PVAT-derived NO in improved NT SV patency, it is suggested that increased NO availability contributes to the improved patency obtained in composite SV-internal thoracic artery Y-grafts. A number of *in vitro* studies using SV graft segments have shown that PVAT possesses anti-contractile effects^[1,11]^. For example, the role of adiponectin in the crosstalk between PVAT and the vessel wall was studied on human SV segments *ex vivo*^[12]^. Here, adiponectin was shown to improve eNOS function by promoting phosphorylation and improving the synthesis of BH4, a critical cofactor necessary for eNOS activity. Also, due to its close proximity to the adventitia, PVAT is ideally placed, not only for a direct effect on adjacent vascular smooth muscle cells but also by transporting adipocyte derived factors, including NO, from the PVAT to the vessel wall via vasa vasorum^[1,11]^. Furthermore, PVAT also provides mechanical support to NT SV grafts, maintaining normal SV architecture, reducing vascular damage at harvesting, and protecting against the effects of arterial hemodynamics after implantation^[6]^. Although interest and use of the NT technique has increased in recent years, different methods aimed at improving graft performance focus on the replacement or repair of the damage inflicted on the SV when using CT harvesting. In particular, the use of various forms of synthetic external support have been described ranging from Dacron® to external metal stents^[13]^. Why should such strategies be necessary when improved SV patency is achieved by preserving normal vessel architecture and PVAT, endotheliumand adipocyte-derived NO?

## CONCLUSION

Certain harmful effects of vascular damage during harvesting the SV on graft patency have been recognised for many years, particularly that to the endothelium. High-pressure saline distension is still used in a high proportion of cases to overcome spasm, a procedure that results in considerable damage to the luminal endothelium and endothelium-derived NO. While a variety of endothelium damage inhibitor solutions are presently used, many surgeons do not consider other sources of NO that are damaged or removed at harvesting. By harvesting the SV intact, it could be said that a Frog can Become a Princess, but without shedding its skin.

## Data Availability

The authors declare that the data supporting the findings of this study are available in the references cited.

## Artificial Intelligence Usage

The authors declare that no artificial intelligence tool was used in the preparation of this article.
